# US regulatory compliance for medical combination products: an overview

**DOI:** 10.3389/fmedt.2024.1486318

**Published:** 2024-12-16

**Authors:** Manav V. Singh, Prafulla Apshingekar, Sanyam Gandhi, Om V. Singh

**Affiliations:** ^1^ Department of Biology, College of Computer, Mathematics, and Natural Sciences, University of Maryland, College Park, MD, United States; ^2^ Indimed Global Limited, Slough, United Kingdom; ^3^ Regulatory Affairs Department, Takeda Pharma, Cambridge, MA, United States; ^4^ Advance Academic Programs, Krieger School of Arts and Sciences, Johns Hopkins University, Washington, DC, United States

**Keywords:** medical device, combination products (CP), Food and Drug Administration (FDA), Office of Combination Products (OCP), Office of Chief Counsel (OCC), Request for Designation (RFD), Federal Food Drug and Cosmetic Act (FFDCA), regulations

## Abstract

This article provides a high-level overview of US regulatory review and approval processes for the growing field of medical combination products (CPs; those merging drugs with devices and/or biological products). US law defines drugs, medical devices, and CPs in specific ways, and the components of a CP are still subject to their respective regulations while combined. The Food and Drug Administration's Office of Combination Products (OCP) oversees the review and classification of CPs, which is based on their primary mode of action. When a manufacturer submits a Request for Designation for a new CP, the OCP conducts a technical and clinical evaluation to identify risks and verify modes of action and therapeutic benefits. Evaluating the safety and efficacy of CPs and their constituent parts can be challenging due to the many potential interactions. However, as innovation continues in the health care landscape and the variety of CPs on the market increases, manufacturers must stay proactive in complying with regulatory standards and keeping their products safe.

## Introduction

Pharmaceutical innovation, particularly in drugs and biologics, has produced a vast array of new medical treatments and technologies in recent decades. Among these, the category of combination products (CPs) refers to therapeutic or diagnostic products that combine drug, biological, and/or device components. For example, one common type of CP approved by the US Food and Drug Administration (FDA) is prefilled syringes of drugs or biologics (1). However, as technology continues to evolve, this category has expanded and become more diverse, with companies developing more and more novel products that blur lines between drugs, devices, and biologics. In 2023, the worldwide market for drug-device CPs was valued at US$138 billion; with current trends in treatments and conditions, it is expected to grow at a compound annual rate of 9% through 2030 (2).

Accordingly, the regulatory landscape for CPs is also evolving. Because there is such a broad range of technological variation possible in CPs, verifying their safety is challenging, and new products can encounter significant difficulties and delays in the approval process. For instance, it may be hard to determine which agency has oversight for a particular product. At the US FDA, a Request for Designation (RFD) or Pre-RFD application can be submitted to receive feedback on how a product should be classified, based on how the product achieves its intended purpose. This article provides an overview of regulatory review and approval processes for CPs in the US, including how product categories are defined, product classification designation, and technical/clinical evaluation, and briefly considers future developments in the CP market.

## Legal definitions and classifications of medical products

While the integration of different technologies into CPs has revolutionized some aspects of health care in the US, it also complicates regulation of these products, and additional compliance steps may be required for manufacturers. When seeking to market medical products in the US, it is essential to first understand how drugs, devices, and CPs are legally defined and categorized.

### Drugs

Section 321(g) of the US Federal Food, Drug, and Cosmetic Act (FFDCA) defines a drug as follows:

(g)(1) The term “drug” means (A) articles recognized in the official United States Pharmacopoeia,1 official Homoeopathic Pharmacopoeia of the United States, or official National Formulary, or any supplement to any of them; and (B) articles intended for use in the diagnosis, cure, mitigation, treatment, or prevention of disease in man or other animals; and (C) articles (other than food) intended to affect the structure or any function of the body of man or other animals; and (D) articles intended for use as a component of any article specified in clause (A), (B), or (C).

### Medical devices

The following section of the FFDCA, 321(h), defines a medical device:

(h)(1) The term “device” … means an instrument, apparatus, implement, machine, contrivance, implant, in vitro reagent, or other similar or related article, including any component, part, or accessory, which is-

(A) recognized in the official National Formulary, or the United States Pharmacopeia, or any supplement to them,

(B) intended for use in the diagnosis of disease or other conditions, or in the cure, mitigation, treatment, or prevention of disease, in man or other animals, or

(C) intended to affect the structure or any function of the body of man or other animals, and

which does not achieve its primary intended purposes through chemical action within or on the body of man or other animals and which is not dependent upon being metabolized for the achievement of its primary intended purposes. The term “device” does not include software functions excluded pursuant to section 360j(o) of this title.

While the act originally became law in 1938, materials acting through chemical means or metabolism in the body were excluded from the definition by the Medical Device Amendment of 1976 to differentiate them from drugs. In 1992, the phrase “any of its principal intended purposes” was revised to “its primary intended purposes” to allow more products to be classified as devices. This change allowed additional products to be categorized under devices (4).

### Combination products

Section 503(g) of the FFDCA established CPs as a distinct class of medical products in 1991. CPs are defined under Title 21 of the Code of Federal Regulation, Section 3.2(e):

(e) Combination product includes: (1) A product comprised of two or more regulated components, i.e., drug/device, biologic/device, drug/biologic, or drug/device/biologic, that are physically, chemically, or otherwise combined or mixed and produced as a single entity; (2) Two or more separate products packaged together in a single package or as a unit and comprised of drug and device products, device and biological products, or biological and drug products; (3) A drug, device, or biological product packaged separately that according to its investigational plan or proposed labeling is intended for use only with an approved individually specified drug, device, or biological product where both are required to achieve the intended use, indication, or effect and where upon approval of the proposed product the labeling of the approved product would need to be changed, e.g., to reflect a change in intended use, dosage form, strength, route of administration, or significant change in dose; or (4) Any investigational drug, device, or biological product packaged separately that according to its proposed labeling is for use only with another individually specified investigational drug, device, or biological product where both are required to achieve the intended use, indication, or effect.

Thus, a CP is a product with constituent parts that include two or more types of regulated products (drugs, biologics, or medical devices), whether they are mixed as one entity, packaged together, or cross-labeled for use only together (5). The various components of a CP are still considered drugs, biologics, or devices and subject to appropriate regulations while combined.

Common CPs include delivery devices filled or coated with drugs or biologics. Drug-biologic combinations such as antibody-drug conjugates are also classified as CPs in the US, though not in other countries. Curiously, another product only considered a CP in the US is a dedicated light source labeled for use with a photoreactive drug. The FDA has established nine categories of CPs (3) that are used in standard product labeling (Table 1).

**Table 1:** FDA categories of combination products^a^.

Type/Description	Common example(s)	Code
Type 0: not a combination product		C112160
Type 1: convenience kit of co-package	Drug and device are provided as individual constituent parts within the same package [Drug or biological product vials packaged with device(s) or accessory kits (empty syringes, auto-injectors, transfer sets), first aid or surgical kits containing devices and drugs]).	C102834
Type 2: prefilled drug delivery device system	Drug is filled into or otherwise combined with the device and the sole purpose of the device is to deliver drug (Prefilled drug syringe, auto-injectors, metered-dose inhalers, dry powder inhalers, nasal-spray, pumps, transdermal systems, prefilled iontophoresis system or microneedle “patch”).	C102835
Type 3: prefilled biologic delivery device system	Biological product is filled into or otherwise combined with the device and the sole purpose of the device is to deliver biological product (Vaccine or other biological product in a prefilled syringe, autoinjector, nasal spray, transdermal systems or microneedle patch pre-loaded with biological product).	C102836
Type 4: device coated/impregnated otherwise combined with drug	Device has an additional function in addition to delivering the drug (Drug pills embedded with sensors, contact lens coated with a drug, drug-eluting stents, drug-eluting leads, condoms with spermicide, dental floss with fluoride, antimicrobial coated catheters/sutures, bone cements with antibiotics).	C102837
Type 5: device coated or otherwise combined with biologics	Device has an additional function in addition to delivering the drug (Live cells seeded on or in a device scaffold, extracorporeal column with column-bound protein [Prefilled syringes (i.e., vaccines in a prefilled syringe), Microneedle patches preloaded with a biologic product, Transdermal patches coated with biologic, Condoms with spermicide, Antimicrobial wound dressings, Bone cement with antibiotics].	C102838
Type 6: drug/biologic combination products	Antibody-drug conjugates [ADCs, e.g., Brentuximab vedotin (Adcetris), Trastuzumab emtansine (kadcyla), and Sacituzumab govitecan (Trodelvy)] and progenitor cells combined with a drug to promote homing [Regenerative medicine products, e.g., Carticel (biologic product for the treatment of cartilage defects), Celution (medical device that extracts cells from adipose tissue)].	C102839
Type 7: separate products requiring cross labeling	Drug -led combination products, Light-activated drugs or biological products not co-packaged but labeled for use with a specific light source device [Photodynamic therapy (PDT) e.g., Porfimer sodium—photosensitizer activated by red light from a laser, Aminolevulinic acid—drug applied to skin and activated by a blue light, Cytalus—imaging drug that illuminates ovarian cancer tissue].	C102840
Type 8: possible combination products based on cross labeling of separate products	Drug/biological product under development utilizes a device, but unclear whether the final product will require that the two be cross-labeled (Microneedles coated with medication to trigger immune system, Light-activated drugs)	C102841
Type 9: other type of Part 3 combination products	Combination products not otherwise described Drug/Device/Biologic Product are combined in a single product (e.g., a prefilled syringe containing an antibody-drug conjugate), device to manufacture a biologic also includes a drug or biologic in the kit, or the product contains two different combination product types (e.g., Type 1 and Type 2 are provided together))	C102842

^a^ Adopted from Combination Product types available online at: https://www.fda.gov/industry/structured-product-labeling-resources/combination-product-types.

## FDA review and designation of CPs

Different centers within the FDA are responsible for regulating and reviewing drugs, biologics, and devices. Products combining different categories can cause difficulties in cases where it is unclear which pre-market approval process should be applied, and which centers should have jurisdiction over the product. The Safe Medical Devices Act of 1990 established a way to designate which agency component should review a particular CP based on its Primary Mode of Action (PMOA) but gave the FDA discretion to determine the most appropriate authority to regulate CPs.

In 2002, the Medical Device, User Fee and Modernization Act created the FDA's Office of Combination Products (OCP) to oversee review of CPs and assign them to Centers. An algorithm that suggests Center assignment based on previously approved similar products and expertise available at the various Centers, but in general the designation is based on PMOA (6). Due to their varying constituent products, CPs typically have multiple modes of action, which must be identified during the classification process. The PMOA is defined as the single mode of action that contributes most to the overall intended therapeutic effects of the CP. The difference between the Mode of Action (MOA) of a drug and a medical device is defined in Sect. 201(h) of the FFDCA, which explains that a product is subject to regulation as a medical device if it does not achieve its effect through chemical or metabolic action within or on the body (7).

**Figure 1:**
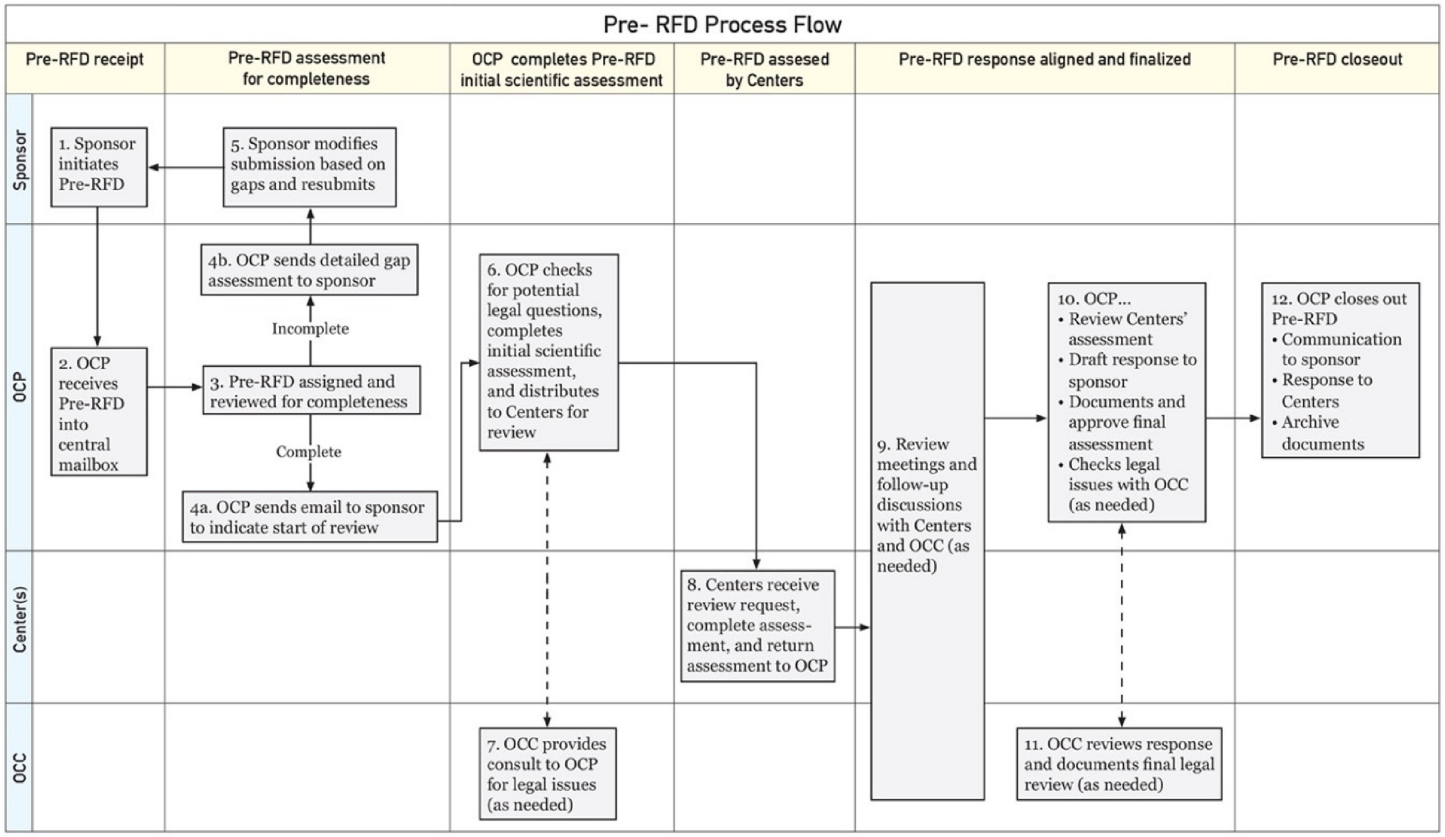
FDA Pre-RFD workflow*. *Adopted from: Nguyen and Sherman (9); OCP: Office of Combination Products; OCC: Office of Chief Counsel.

The FDA also offers a Pre-Request For Designation (RFD) workflow for manufacturers to seek agency feedback on the potential designation of a medical product (Figure 1). It is less formal and more interactive than the official RFD process and can be used during product design or any stage of product development to help in decision making (8, 9). The Pre-RFD requires less supporting material (for example, the manufacturer does not need to propose a designation and justify it with a rationale or discuss other products they have on the market). The agency reviews the Pre-RFD submission and provides feedback on their initial thought process around classifying the product.

To provide further help for CP manufacturers, the OCP publishes guidance documentation on product classification and CPs. Product-specific guidance is also offered by the various centers, including the Center for Drug Evaluation and Research (CDER), Center for Biologic Evaluation and Research (CBER) and Center for Device Radiological Health (CDRH). These guidance documents are listed in Table 2. Further, Table 3 identifies device product codes (procodes) for device constituent parts of Abbreviated New Drug Application (ANDA), New Drug Application (NDA) and Biological License Application (BLA) combination products.

**Table 2:** Guidance on combination products from CBER, CDER, and CDRH^a^.

Category	FDA guidance	Date released
Pre-market	Purpose and content of use-related risk analyses for drugs, biological products, and combination products	July, 2024
	Essential drug delivery output (EDDO) for device intended to deliver drugs and biological products	June 2024
	Regulatory considerations for prescription drug use-related software	September 2023
	Application of human factors engineering principles for combination products: questions and answers	September 2023
	Principles of premarket pathways for combination products	January 2022
	Requesting food and drug administration feedback on combination products	December 2020
	Technical considerations for demonstrating reliability of emergency-use injectors submitted under a BLA, NDA or ANDA	April 2020
	Bridging for drug-device and biologic-device combination products	December 2019
	Technical considerations for pen, jet, and related injectors intended for use with drugs and biological products	June 2013
	Glass syringes for delivering drug and biological products: technical information to supplement international organization for standardization (ISO) standard 11040-4	April 2013
	New contrast imaging indication considerations for deices and approved drug and biological products	December 2009
	Early development considerations for innovative combination products	September 2006
	Application user fees for combination products	April 2005
	Submission and resolution of formal disputes regarding the timeliness of premarket review of a combination product	May 2004
Post-market	Post marketing safety reporting for combination products	July 2019
	Compliance policy for combination product post marketing safety reporting	April 2019
	Current good manufacturing practice requirements for combination products	January 2017
	Submissions for postapproval modifications to a combination product approved under a BLA, NDA, or PMA	Jan 2013
Jurisdictional	How to prepare a pre-request for designation (Pre-RFD)	February 2018
	Classification of products as drugs and devices and additional product classification issues	September 2017
	How to write a request for designation (RFD)	April 2011
	Deices used to process human cells, tissues, and cellular and tissue-based products (HCT/Ps)	July 2007

^a^ Adopted from: Combination Products Guidance Documents at https://www.fda.gov/combination-products/guidance-regulatory-information/combination-products-guidance-documents (Last accessed August 14, 2024).

**Table 3:** Device product codes for device constituent parts of aNDA/NDA/BLA CPs^a^.

Product codes	Device constituent part	Product description	Classification	Regulations number
FMF	Syringe	Syringe, Piston	2	880.5860
MEG	Syringe with injury prevention features	Syringe, Antistick	2	880.5860
KZH	Autoinjector	Introducer, Syringe Needle	2	880.6920
KZE	Needle-free injector, jet injector	Injector, Fluid, Non-Electrically Powered	2	880.5430
IQG	Syringe holder	Adaptor, Holder, Syringe	1	890.5050
KCO	Ear, nose, and throat drug administration device	Nasal spray, ENT delivery	1	874.5220
QIY	Nasal spray for systemic delivery	Nasal spray, systemic delivery, CDER or CBER led	Not classified	–
CAF	Nebulizer	Nebulizer	2	868.5630
FPA	IV administration sets/kits	Set, Administration, Intravascular	2	880.5440
FPK	IV tubing	Tubing, Fluid Delivery	2	880.5440
KPE	IV bag	IV Container	2	880.5025
KZD	Pressure infusor for IV bags	Infusor, Pressure for IV Bags	1	880.5420
LHI	IV transfer set	Set, I.V. Fluid Transfer	2	880.5440
ONB	Vial adapter	Closed antineoplastic and hazardous drug reconstitution system	2	880.5440
KYW	Medicine cups	Container, Liquid Medication, Graduated	1	880.6430
KYX	Oral syringes, droppers	Dispenser, Liquid medication	1	880.6430
FRN	Infusion pumps	Pump, Infusion	2	880.5725
LZG	Insulin infusion pumps	Pump, Infusion, Insulin	2	880.5725
MRZ	Infusion pump accessories	Accessories, Pump, Infusion	2	880.5725
HGD	Vaginal applicators	Applicator, Vaginal	1	884.4520
KDC	Disposable manual surgical instruments (scalpels, clamps, etc.)	Instrument, disposable, surgical	1	878.4800
MQX	Acupuncture needle	Needle, Acupuncture, single use	2	880.5580
QIZ	Implantable material for controlled release	Material, implantable for controlled release, CDER or CBER-led	Not classified	–
QKS	Metered dose inhaler and dry powder inhaler	Inhaler, Metered Dose or Dry Powder, CDER or CBER-led	Not classified	–
QLF	On-body injector	On-Body Injector	2	880.5860
QLH	Intravaginal system for controlled release of drug substance	System, Intravaginal, for Controlled Release of Drug Substance, CDER or CBER-led	Not classified	–
QLI	Vaporizer	Vaporizer, CDER or CBER-led	Not classified	–
NSC	Pen injector	Injector, pen	2	880.5860
LZH	Enteral infusion pump	Pump, Infusion, enteral	2	880.5725
FMI	Hypodermic needle	Needle, hypodermic, single lumen	2	880.5570
FPB	Infusion line filter	Filter, infusion line	2	880.5440
NVO	MDI and nebulizer spacer	Spacer, direct patient interface	2	868.5630
HDT	IUD introducer	Device, intrauterine, contraceptive and introducer	3	884.5360

^a^ Device product codes (procodes) for device constituent parts of ANDA/NDA/BLA combination products, Available online at: https://www.fda.gov/combination-products/device-product-codes-procodes-device-constituent-parts-andandabla-combination-products, accessed August 14, 2024.

When a CP manufacturer submits a formal RFD, the FDA follows a 60-day timeline to review the submission, evaluate the product, and respond. The OCP conducts an initial scientific evaluation, followed by further assessment from the centers. Meetings are held with the centers and the Office of Chief Counsel if necessary to consult about the proper jurisdiction for the product, and the OCP decides based on their responses. The OCP then informs the manufacturer of the decision. A manufacturer can request that the agency reconsider their response within 15 days of the decision; however, the final response is made, and the RFD is closed within 90 days of the initial submission.

## Technical and clinical evaluation of CPs

The primary reason for FDA review of medical products is determining whether they are safe and effective for their intended therapeutic purpose. Therefore, CPs go through a technical and clinical evaluation process to identify risks and verify modes of action and therapeutic benefits. Medical devices that come in contact with the human body are also required to meet the international standards for biocompatibility defined in ISO 10993.

If the individual constituent parts of a CP have already been approved by the appropriate regulatory agency, their risks are known. However, if a manufacturer introduces new components or new modes of action, indications, target populations, or methods of use for previously approved ones, the new risks must be assessed by appropriate Centers (i.e., CDER/CDRH/CBER) assigned by OCP.

Evaluating CPs and their components poses an additional challenge due to the many potential interactions. For example, while an antibiotic drug could be added to an implant device for an antibacterial effect (10, 11), antibiotics present an associated risk of inducing resistance (12). Also, if a device is used to carry a drug, the materials, production processes, or storage environment for the device component could affect or interfere with the activity of the drug. Device materials such as carrier polymers may need their own drug safety evaluation. CP structural design and carrier materials can also potentially affect rates of drug release in the body, which must be accounted for in device-drug CPs. If the CP is sterilized after assembly, the effects of sterilization on drug components are another consideration.

Conversely, drug components can have unintended effects on device components: for example, in device-drug CPs such as oxygenators with heparin, sutures with triclosan, and stents with paclitaxel (13–15), the drug dosage used and loading processes may alter the devices’ mechanical and surface properties (16). Drugs in devices may also affect the biocompatibility of the devices and the results of biological evaluation tests.

Before the regulatory evaluation, the manufacturer of a CP should have conducted clinical trials that comply with international quality control standards (17). They should fully understand and be able to demonstrate the modes of action and PMOA of their product as well as the intended benefits, risks, and possibility of adverse events in use. Components may require preliminary studies before trials of the CP: for example, the pharmacokinetics and metabolism of drugs must be characterized (18) and their safety tested if they have not been previously approved. When all these aspects of a new CP have been sufficiently studied by the manufacturer, the FDA is better able to evaluate it.

Medical product regulation exists to ensure that products are scientifically and comprehensively evaluated for safety and efficacy. As a result, the technical and clinical review process is rigorous, and CPs are subjected to systematic assessment on various levels by the appropriate agencies before they are approved for the U.S. market. The FDA's regulatory procedures have been described here, but agencies in other countries also have their own evaluation processes for drugs, devices, and CPs.

## Risk management and post marketing safety reporting

As an ongoing process, the risk management is required to be maintained throughout the lifecycle of CP. The risk management relates to proactive identification of hazards and harms, risks evaluation, mitigating and controlling of risks. In CPs, the integrated risk management process involves drug and device related risk vs. benefits. These risks can be identified based on Quality Risk Management (ICH Q9) and Medical Device Risk Management (ISO 14971). Standards, i.e., AAMI TIR105:2020) (19) and ISO 14971 (ISO TR 24971:2020) (20), are helpful references to determine risk assessment and management of drug-device CPs. Kumoluyi and Khanolkar (21) discussed the relationship between risk management and lifecycle management processes. To evaluate the risk and their mitigation, the design, manufacturing process, purchasing, and management needs to be among top level control processes. Risk management documents are required to be updated throughout the lifecycle of CPs due to new data acquisition based on post-market surveillance.

FDA issued final role on postmarketing safety reporting (PMSR) requirements for CPs on Dec. 20, 2016 (81 FR 92603) and codified under 21CFR Part 4, Subpart B). These PMSR requirements are for CPs that have received FDA clearance/approval. FDA provided guidance document entitled “Postmarketing Safety Reporting for combination products” in July 2019 to Industry and FDA staff, explaining PMSR final rule, PMSR report types, timeline and process consideration of PMSR, and scenarios to comply with CPs on PMSR requirements.

## Perspectives on future CP developments

Medical science is constantly producing innovations in health care technology and treatment methods. CPs are far from an exception: changes and improvements are likely to continue in CP technology, design and manufacturing, and the important area of risk management and post-market surveillance.

CPs are on the cutting edge of technological developments that extend possibilities in health care. Today, the product category includes drug-eluting stents and other implants, prefilled and wearable drug delivery devices, and “smart” digitally connected devices such as inhalers, among many other emerging technologies. Software integrations and enhancements of medical CPs are a likely area of expansion in the future. Apps and digital health monitors already help people track and personalize their treatment, and more advanced technology, such as AI, may make these solutions more effective and further improve the experience for patients and their doctors.

CPs also tend to present more design challenges than individual drugs, devices, or biologics, as they incorporate different types of components that must work together. Improving design and production strategies will be essential for manufacturers, based on input from engineers, regulatory experts, health care professionals, and end users. Seeking feedback and collaborating with these stakeholders can help refine designs and yield strategies that ensure CPs are safe, effective, and easy to use.

The third piece of the puzzle for CP innovations is managing risk and tracking performance throughout the life cycle of these products. Greater complexity in design leads to a wider range of possible risks and unforeseen interactions. As the number and variety of CPs on the market increase, per FDA's guidance (Table 2) manufacturers must deploy risk control strategies and post-market surveillance to stay proactive in complying with regulations and keeping their products safe.

## Conclusion

The regulatory environment for medical products in the US is complex and presents particular challenges when it comes to CPs, as they combine types of products that are regulated differently. This article outlined key definitions, processes, and stages in the FDA approval process that are relevant to manufacturers of drug-device combinations and other CPs. In addition to staying aware of the compliance requirements for CPs, manufacturers should follow regulatory guidance, implement high-quality systems and manufacturing practices, and follow up with risk management to ensure product safety.

Both innovation and compliance are essential for advancing the CP field. By observing regulatory standards while developing novel technologies, manufacturers can continue to market safe and effective products that revolutionize treatments and user experiences, to their own benefit and that of end users.
